# Supplementary material for: Spatiotemporal distribution analysis of syphilis in Brazil: Cases of congenital and syphilis in pregnant women from 2001–2017
Source: PLoS One. 2022 Oct 6;17(10):e0275731. doi: 10.1371/journal.pone.0275731 (PMC9536537; doi:10.1371/journal.pone.0275731)
Supplement: Attachment — Submitted filename: Review.pdf [file pone.0275731.s007.pdf]

The article presents a relevant topic using novel data with sub-national and temporal detail, potentially advancing the current understanding of CI and SiP's geographical diffusion and temporal evolution. I have read it with great interest and enjoyed the manuscript in general. However, there is room for improvement in various parts, in particular: the literature review should be extended, the data section needs to be more detailed, the methods should include spatial econometric techniques, and the results and discussion sections should better elaborate on the implications of the study results and their impact on current research .

Major revisions:

1. The references need to be formatted properly (a lot of the journal names are shortened), and often the article or volume number is missing. E.g. reference number 35 should be: Domingues RM, Szwarcwald CL, Souza PR, Leal MD. Prevalence of syphilis in pregnancy and prenatal syphilis testing in Brazil: birth in Brazil study. Revista de saúde pública. 2014;48(5): 766-74. In one occasion you cite WHO and others World Health Organization, it should be consistent throughout the references. Reference number 10 has the authors' names listed in capital letters.
2. The literature is missing a few important publications, their careful reading and inclusion will definitely improve the manuscript. See for instance, but not exclusively Marques dos Santos, M., Lopes, A. K. B., Roncalli, A. G., & Lima, K. C. D. (2020). Trends of syphilis in Brazil: a growth portrait of the treponemic epidemic. *Plos one*, *15*(4), e0231029. Rgjh; Soares, K. K. S., Prado, T. N. D., Zandonade, E., Moreira-Silva, S. F., & Miranda, A. E. (2020). Spatial analysis of syphilis in pregnancy and congenital syphilis in the state of Espírito Santo, Brazil, 2011-2018. *Epidemiologia e Serviços de Saúde*, *29*.
3. The paper does not present any reference to the spatial nature of the data from an econometric point of view. It would be useful to add Global and Local Moran's I (or similar indexes) to measure the non randomness of spatial clusters, especially using LISA plots and bivariate LISA plots. See for instance: Brooks, M. M. (2019). The advantages of comparative LISA techniques in spatial inequality research: Evidence from poverty change in the United States. *Spatial Demography*, *7*(2), 167-193.; Yin, F., Feng, Z., & Li, X. (2012). Spatial analysis of primary and secondary syphilis incidence in China,

2004-2010. *International journal of STD & AIDS*, *23*(12), 870-875, Graepp Fontoura, I., Lima, V. C., Fontoura, V. M., Santos, F. S., de Jesus Costa, A. C. P., de Oliveira, F. J. F., ... & Santos Neto, M. (2021). Spatial analysis of congenital syphilis in a federative unit in northeastern Brazil. *Transactions of The Royal Society of Tropical Medicine and Hygiene*, *115*(10), 1207-1217. And more in general: Anselin, L. (2001). Spatial econometrics. *A companion to theoretical econometrics*, *310330*.

4. In the abstract, you mention that CS and SiP is more prevalent among mixed race individuals (newborns and women) but on p.8 the picture is different, and it is described to vary according to macro-regions. Different regions in Brazil have different ethnic compositions, you should make that clear earlier on.
5. Moreover, in the legend in Figures 3 and 4, you report the classifications as translated from Portuguese, which sounds off in English. The authors might want to revise and update that. I suppose 'Brancos' would be individuals of European descent, 'Amarelos' would be of East Asian descent, etc.
6. The Study design section would benefit from a more thorough description of the ethnic categories used. Mixed race is for any mixed race, as in anyone who does not identify as either of black, white, indigenous or East Asian descent?
7. In fig 3 and 4 you present bar charts over Brazil's macro regions map. However, a lot of information is missed because of charts resizing to fit within each macro-region. I would arrange the bar charts without the map, and add for each macro-region the population composition (the main group). That way, it'd be easier to interpret CI and SiP prevalence in said category, whether it is just representative of the population composition or of a disadvantaged population group (I believe the South is predominantly white so it's not a shocker that most CI children are white). One example that comes to mind is the share of Covid deaths among Alaskans, where 30% of Covid19-related deaths were registered among Alaskan Natives, although they represent 15% of the total Alaska population. (https://coronavirus.jhu.edu/data/disparity-explorer). In figure 4: Years spent in education
8. In the spatiotemporal description of results the regions mentioned may not be familiar with audiences outside of Brazil. Are those mainly rural, urban, tropical or large cities?

9. The results and discussion can be improved by contextualising them. For instance, you mention Cassilandia, which is a small city at the border with Mato Grosso: elaborate on why its results are relevant when compared to other cities.
10. How does under-reporting, and its decrease in the last 15 years affect the results and its interpretation? Are there population subgroups most likely to be missed?

Minor revision:

11. The article needs careful revision, there are typos throughout the text, as well as some expressions that should be improved (see below a few examples).
12. P3. line 60: condom-less sex (unprotected intercourse?)
13. P.3 line 62 space before full stop
14. P.3 line 67 parenthesis misplaced
15. Page 4 ends with a title
16. Line 188: you don’t mention the male to female ratio for the general pop.
17. L. 201: suggesting a lack of protection against CS : lack of prenatal screening? Or condom use?
18. L. 211: you only mention here that race is self reported, you should mention this in the data section as well
