# Supplementary material for: Spatiotemporal distribution analysis of syphilis in Brazil: Cases of congenital and syphilis in pregnant women from 2001–2017
Source: PLoS One. 2022 Oct 6;17(10):e0275731. doi: 10.1371/journal.pone.0275731 (PMC9536537; doi:10.1371/journal.pone.0275731)

Ministério da Saúde

FIOCRUZ
Fundação Oswaldo Cruz
Instituto Gonçalo Moniz

Salvador-Bahia, Brazil - September 2022

Dear Dr. Laganà,
Editor Plos One

We thank you and the reviewers again for your thoughtful suggestions that helped improve our manuscript. We believe that this revised manuscript is a better and more balanced representation of our research, and we hope that it is now suitable for publication in your journal. The answers to the questions can be found below (PONE-D-21-31051).

Sincerely,

Dr. Fred Luciano Neves Santos
corresponding author
fred.santos@fiocruz.br

Ministério da Saúde

FIOCRUZ
Fundação Oswaldo Cruz
Instituto Gonçalo Moniz

Author's Reply to the Review Report (Reviewer 1)

Reviewer #1: I am overall happy with the changes and the improved manuscript. I have a minor (yet very important) comment with regards to the graphs presented in the appendix of the manuscript.

Question 1 - Reviewer #1. Fig. 3: The 'yellow' category in the legend has been replaced with a much more appropriate 'Asian ancestry'. However, the graph title is 'self reported skin color', a category that has been outdated for a while. Self reported race or ethnicity is more appropriate than skin color.

Reply: We thank the reviewer for bringing this error to our attention. We have updated the graph title and changed the Fig. 3 as follows:

Before:

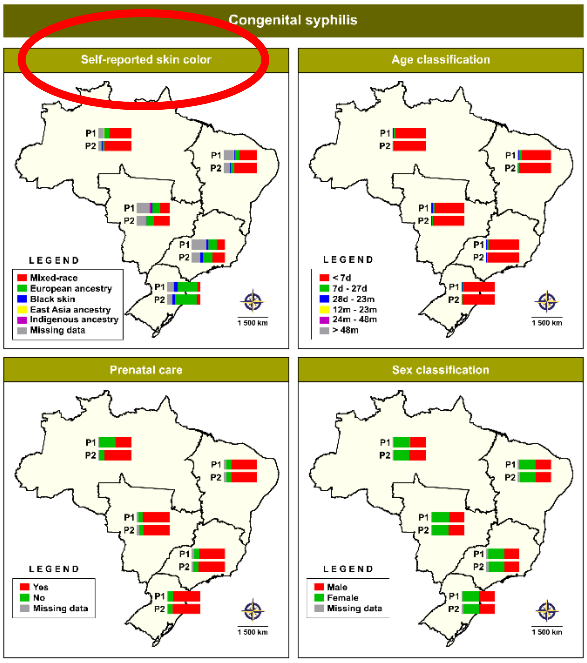

After:

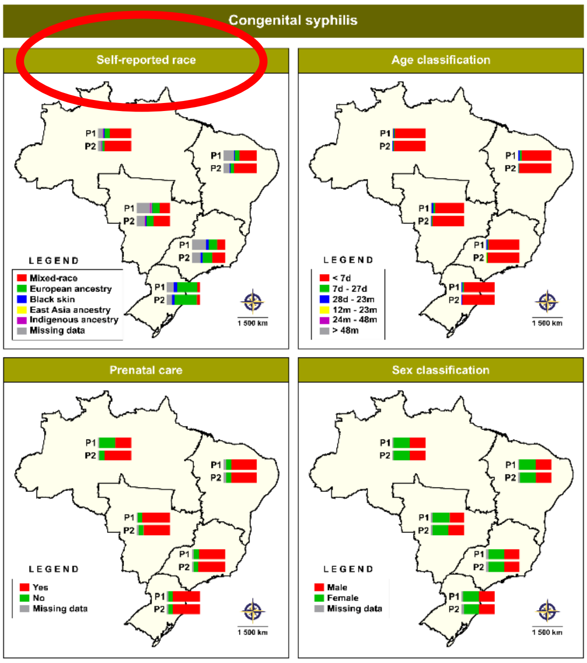

Rua Waldemar Falcão, nº 121, Candeal, Salvador, Bahia, CEP 40296-710, Brasil
Tels. +55 71 3176-2322; +55 71 99390-3004
E-mail: fred.santos@fiocruz.br

Question 2 - Reviewer #1. Fig. 4: still includes 'yellow' in the legend..

Reply: We thank the reviewer for this observation We have updated the Fig. 4 as follows:

Before:

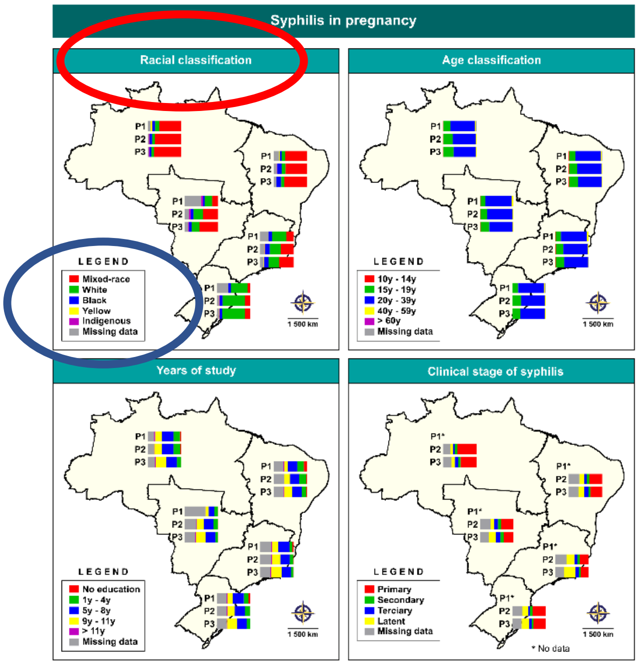

After:

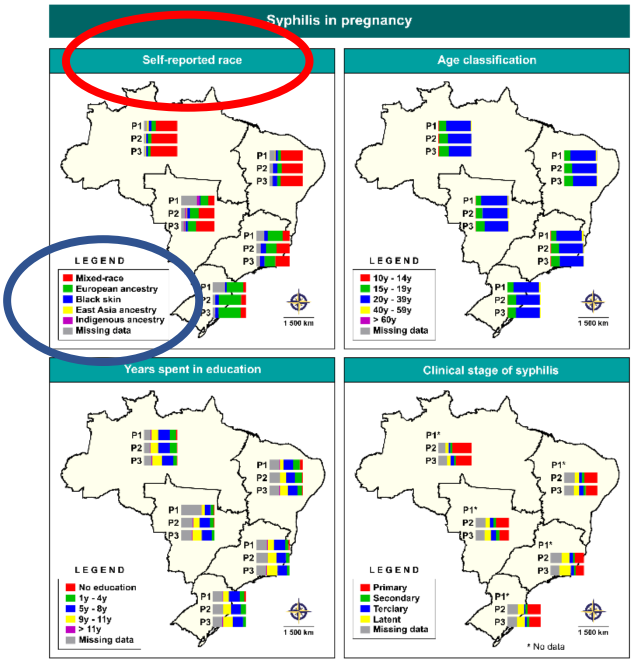

Supplement: Attachment — Submitted filename: Reply_PONE-D-21-31051R2.pdf [file pone.0275731.s010.pdf]
