# Supplementary material for: Spatiotemporal distribution analysis of syphilis in Brazil: Cases of congenital and syphilis in pregnant women from 2001–2017
Source: PLoS One. 2022 Oct 6;17(10):e0275731. doi: 10.1371/journal.pone.0275731 (PMC9536537; doi:10.1371/journal.pone.0275731)
Supplement: Attachment — Submitted filename: Comments on the PloS One Brazil CS and Maternal 15Feb2022.docx [file pone.0275731.s008.docx]

Reviewer Comments: ‘Spatiotemporal distribution analysis of syphilis in Brazil’

February 15, 2022

1. Major comment: Line 37 Abstract and in methods, results of paper. The WHO global estimates of CS use the number of live births as the denominator for the calculation of rate of congenital syphilis. In order to have these results considered at the regional and global levels, use of the global case rate definition would be of greater value. Also the WHO criteria for recognizing national elimination of mother to child transmission of syphilis (EMTCT) is <50 cases/100,000 ‘live births’. This analysis could contribute “directly” to Brazil’s national and provincial monitoring and surveillance of SiP and CS towards EMTCT if the rates of CS were calculated according to global case rate definitions.
   a. <https://www.who.int/publications/i/item/9789240039360>
   b. <https://journals.plos.org/plosmedicine/article?id=10.1371/journal.pmed.1002329>
   c. <https://www.who.int/reproductivehealth/congenital-syphilis/WHO-validation-EMTCT/en/>
   d. <https://www.who.int/reproductivehealth/congenital-syphilis/emtc-gvac/en/>
2. Abstract line 45-46, please provide the values that increased 4000%... from what to what?
3. Introduction line 54, need to use the most recent WHO estimates for syphilis
   a. <https://pubmed.ncbi.nlm.nih.gov/31384073/>
   b. Rowley J, Vander Hoorn S, Korenromp E, et al. Chlamydia, gonorrhoea, trichomoniasis and syphilis: global prevalence and incidence estimates, 2016 *WHO Bulletin.* 2019; 97:548-562.
4. Introduction line 56, need to use the most recent WHO estimates for **congenital** syphilis
   a. <https://pubmed.ncbi.nlm.nih.gov/30811406/>

Korenromp EL, Rowley J, Alonso M, et al. Global burden of maternal and congenital syphilis and associated adverse birth outcomes – estimates for 2016 and progress since 2012. *PLOS One*. 2019. 14(2): e0211720.

5. Introduction: Line 63, would delete “following direct contact with bacterium by the mother”
6. Lines 76-86. Please see the proposed edited language for better clarity and consistency with other published references on maternal and congenital syphilis terminology.

*“According to the Brazil MoH, women diagnosed with syphilis during pregnancy, at delivery and / or puerperium should be reported as SiP. This case definition includes symptomatic or asymptomatic pregnant women with at least one reactive syphilis test, either treponemal or nontreponemal (of any titer), and without previous recorded syphilis treatment. The case definition of CS includes: all newborns, stillbirths or abortions from women diagnosed with syphilis that have not been treated or who received inadequate treatment, children under 13 years of age with clinical manifestations, radiographic or radiological alterations and reactive nontreponemal or treponemal syphilis tests, and children, products of abortion or stillbirth with biopsy or necropsy microbiological evidence of T. pallidum infection in a sample of nasal discharge or skin lesion, detection of T. pallidum by means of direct exams by microscopy (dark field or with colored material) [13].”*

7. Lines 88-89. Please provide appropriate references for the statements regarding increases in syphilis. Reference 14 is from 2015 and likely reflects data from several years prior. Consider using the WHO global estimates of syphilis (Rowley et al. described earlier) and the US CDC 2019 surveillance report <https://www.cdc.gov/std/statistics/2019/default.htm> for the US as current references.
8. Methods: please describe in more detail the joinpoint method of identifying the year intervals of analysis.
9. What does a skin color of ‘yellow’ imply? The skin colors are difficult to interpret without the addition of information related to culture, origin or ethnicity. (Asian, Hispanic, Aboriginal, African etc). “Mixed-race” is used but it is unclear how the other races can be derived from skin color alone. If alternatives or co-naming (e.g. yellow = Asian decent) are not possible would explain the historical context of this color-based naming.
10. Discussion: line 292. The use of “tendency” is unclear. Consider rate of case number
11. Discussion Lines 296-299. Would consider describing Rede Cegonha later in the discussion alongside penicillin shortages
12. Discussion line 295: instead of “syphilis remains a national public health concern” consider that “syphilis should be “prioritized” as a national public health concern due to the dramatic increases in rates of SiP and CS”. This would be a good ending for the first paragraph.
13. The discussion is quite long and repeats information about gender of babies and race of mothers. Would recommend shortening and removing redundancy.
14. Line 315, would reference global estimates of CS by Korenromp et al stated comment number 4.
15. Line 320 needs a reference for global BPG shortages. Suggest this reference which highlights Brazil. Nurse-Findlay S, et al Françoise Bigirimana F, Ouedraogo L, Pyne-Mercier L. Supply, Demand, and Shortages of Benzathine Penicillin for Treatment of Syphilis: A Market Assessment. PLoS Medicine. 2017;14 (12):e1002473.
16. Line 345 would consider the Brazilian reference Rocha AFB et al. Complications, clinical manifestations of congenital syphilis and aspects related to its prevention: an integrative review. <https://pubmed.ncbi.nlm.nih.gov/34287560/>
17. Discussion lines 326 -321 from “A similar prevalence… “ Would delete this phrase as it is well known that the syphilis does not preferentially affect the gender of the infant.
18. Recommend other areas to shorten for example: “With respect to self-reported skin color, the majority of reported CS cases occurred among newborns identified by the family as ‘mixed-race’. Other data have also shown that the incidence of congenital syphilis tends to be significantly higher in black or ‘mixed-race’ children. In fact, several studies have linked cases of CS with family history, including the racial classification of children’s parents, with high numbers of pregnant women self-identifying as ‘mixed-race’ or black [22–25].”……….Can be shortened to: “The majority of reported CS cases occurred among newborns identified by the family as ‘mixed-race’ similar to other reports [22-25]”. Shortening the discussion to the relevant details will ensure more people read it.
19. Citation 32 would change to the global guidelines <https://www.who.int/reproductivehealth/publications/rtis/syphilis-ANC-screenandtreat-guidelines/en/>
20. The paragraphs starting on lines 380 and 394 are nearly identical in content and interpretation. Would delete one of these paragraphs. There are multiple examples of this type of duplication in the discussion. As much as possible, would recommend that the authors stay close to the findings reflected from their spatiotemporal analysis with aa greatly shortened summary of the literature that contributes to the understanding of risk factors and other contributors to increasing syphilis diagnoses in Brazil.
